# The relationship between psychological flexibility and health locus of control in patients after total hysterectomy: a latent class analysis

**DOI:** 10.3389/fpsyg.2025.1662370

**Published:** 2025-12-02

**Authors:** Wantian Liang, Wenli Liang, Danni Ou

**Affiliations:** 1 Department of Nursing, Guangdong Pharmaceutical University, Guangzhou, China; 2 Department of Continuing Education, Guangdong Pharmaceutical University, Guangzhou, China; 3 Department of Joint Surgery, Guangzhou First People's Hospital, Guangzhou, China

**Keywords:** total hysterectomy, psychological flexibility, health locus of control, latent class analysis, postoperative mental health, nursing

## Abstract

**Objectives:**

This study aimed to examine the latent classes of psychological flexibility in patients after total hysterectomy and to analyze the characteristics of each class as well as their associations with health locus of control.

**Methods:**

A total of 214 hospitalized patients who underwent total hysterectomy at a tertiary maternal and child healthcare hospital in mainland China were surveyed between April 2022 and May 2023. Latent class analysis was conducted to identify distinct psychological flexibility classes. Logistic regression and one-way Analysis of Variance (ANOVA) were used to explore differences across classes in sociodemographic variables and health locus of control scores.

**Results:**

Three latent classes of psychological flexibility were identified: Inflexible Class (*n* = 23, 10.75%), Moderate Class (*n* = 144, 67.29%), and Flexible Class (*n* = 47, 21.96%). Patients with children were significantly more likely to belong to the Moderate Class (OR = 18.920, *p* = 0.033); patients under age 45 were more likely to be in the Inflexible Class (OR = 0.028, *p* = 0.016); and those with a monthly per capita household income ≥ 5,000 CNY were more likely to fall into the Flexible Class (OR = 3.795, *p* = 0.027). Significant class differences were found in internal health locus of control (*F* = 5.649, *p* < 0.05) and chance health locus of control (*F* = 30.810, *p* < 0.05).

**Conclusion:**

Psychological flexibility in patients after total hysterectomy shows heterogeneity. Healthcare providers should carefully assess the characteristics of each psychological classes and implement tailored interventions to support patients’ mental well-being and postoperative recovery.

## 1 Introduction

Total hysterectomy, often referred to as “surgical menopause,” is the second most common gynecological surgery after cesarean section (Pickett et al., 2023) and is among the most frequently performed procedures in non-pregnant women (Querleu et al., 2024). As a therapeutic approach, total hysterectomy is widely used to treat various gynecological conditions such as uterine fibroids, endometriosis, uterine prolapse, cervical cancer, and ovarian malignancies. Globally, it is a commonly adopted surgical intervention. Epidemiological data indicate that in China, the number of annual total hysterectomy procedures increased from over 1 million in 2005 to more than 2.8 million by 2016 (Ma et al., 2021). In Switzerland, Germany, and Canada, the procedure rates are approximately 283, 257, and 243 per 100,000 women, respectively (Stoller et al., 2020; Baffour Awuah et al., 2024; Chen et al., 2021). In the United States, over 600,000 women undergo total hysterectomy each year (Harvey et al., 2022).

The uterus is a central organ of the female reproductive system and is often viewed as a symbol of femininity, sexuality, fertility, and motherhood (Mahardika et al., 2021). As an organ-removal procedure, total hysterectomy not only results in the loss of reproductive capacity but can also lead to significant physiological and psychological changes (Li et al., 2023). Physiologically, patients may experience pelvic floor dysfunction (Chang et al., 2025), ovarian insufficiency (Wallin et al., 2019), and reduced sexual function (Till et al., 2022). Psychologically, they may suffer from a disrupted self-concept (Goudarzi et al., 2021) and develop negative emotional responses such as depression, anxiety, stress, and even post-traumatic stress disorder (PTSD), all of which severely affect overall well-being and quality of life. Studies have shown that 45.5% of patients experience depressive symptoms following total hysterectomy (Ghali et al., 2025), while 68.1 and 85.6% report symptoms of anxiety and stress, respectively (Al-Amer et al., 2025). Additionally, 16.4% develop PTSD (Casarin et al., 2022). These psychological consequences are particularly pronounced in women who still have reproductive desires (Li et al., 2023). Therefore, identifying modifiable psychological factors that can buffer against these adverse outcomes is of critical importance for improving patients’ postoperative well-being.

In this context, psychological flexibility (PF) emerges as a crucial psychological construct. Rooted in the Acceptance and Commitment Therapy (ACT) (Hayes, 2004), psychological flexibility refers to an individual’s capacity to remain psychologically present and to act in ways that align with personal values, even in the presence of distressing thoughts or emotions (Cherry et al., 2021). It represents a key mechanism for adaptive psychological functioning. Individuals with high levels of psychological flexibility are capable of self-regulation based on internal values and can flexibly navigate challenges while maintaining coherence between thought and behavior (Gentili et al., 2019). Studies have reported that enhancing psychological flexibility can alleviate stress, anxiety, and depression while improving overall well-being and life satisfaction (Dawson and Golijani-Moghaddam, 2020). Consequently, it is plausible that psychological flexibility may serve as a vital protective factor for patients coping with the psychological trauma of hysterectomy.

From the perspective of the post-traumatic growth model, an individual’s cognitive evaluation of a traumatic event is shaped by their personal resources (Tedeschi et al., 2018). Psychological flexibility regarded as a key mechanism within ACT, is shaped by internal personal resources, particularly in the context of hysterectomy as a major life stressor (Cyniak-Cieciura, 2021). In Rotter’s social learning theory, locus of control (LOC) is conceptualized as one such personal resource, reflecting an individual’s attributional style regarding outcomes (Rotter, 1966). Locus of control is generally divided into internal and external types: individuals with an internal locus of control attribute outcomes to their own actions, whereas those with an external locus of control attribute outcomes to outside forces (Rotter et al., 1972). Building on this theory, Wallston et al. (1978) proposed the concept of health locus of control (HLC), which refers to individuals’ beliefs, attitudes, and responses when facing adverse health-related situations. Health locus of control comprises three dimensions: internal health locus of control (IHLC), which reflects the belief that one’s health is determined by personal behavior; powerful others health locus of control (PHLC), which reflects the belief that health is determined by influential figures such as medical professionals or family members; and chance health locus of control (CHLC), which reflects the belief that health is determined by fate, luck, or spiritual forces (Wallston et al., 1978). Churchill posited that trauma can activate different types of health locus of control, which may explain or predict individuals’ psychological and behavioral responses (Churchill and Smyth, 2022).

While psychological flexibility has been explored in other clinical populations such as those with chronic pain or cancer, its role in patients following total hysterectomy remains understudied. Furthermore, existing studies predominantly use total scale scores of psychological flexibility, which assume population homogeneity and mask underlying subgroups (Plys et al., 2023). Therefore, it is necessary to conduct research on psychological flexibility of patients following total hysterectomy on the group characteristics level. To achieve this, Latent class analysis (LCA) can be used to identify optimal classification and diverse characteristics in psychological flexibility of patients following total hysterectomy, determined by model fit indices. In our study, we did not use traditional methods such as sensitivity and specificity analyses to explore the simple cut-off value of the total psychological flexibility scores. These approaches often assume all individuals are similar and may overlook important differences between them. As a result, they may not capture the complexity of psychological flexibility in patients after total hysterectomy. In contrast, LCA is a data-driven, person-centered method that identifies naturally occurring subgroups based on patterns in the data, rather than relying on preset thresholds. Additionally, LCA can capture complex group characteristics that are difficult to detect with traditional methods, revealing both similarities and differences between latent classes (Lanza, 2016). These group characteristics will provide a more targeted theoretical basis for developing intervention measures tailored to different groups of patients following total hysterectomy. This statistical method has been widely used in health psychology study research. Previous studies encompassing diverse populations have used the LCA method to classify psychological flexibility into different latent classes. For example, Zhao et al. (2024) identified three classes in a sample of 805 nurses in China: toughness-flexible (32.8%), power-deficit-emotional (23.1%), and toughness-rigid (44.1%).

Therefore, LCA was proposed to determine the optimal latent class on psychological flexibility of patients following total hysterectomy based on the model fitting indicators in our study. It further examines the demographic factors associated with each class and explores the relationship between psychological flexibility and health locus of control. The findings aim to inform targeted strategies to enhance psychological flexibility and provide an empirical foundation for developing personalized psychological interventions in postoperative care.

To guide the current study, we propose the following hypotheses: (1) there are different potential categories of psychological flexibility in patients after total hysterectomy; (2) there will be significant differences in the latent classes of psychological flexibility among patients after total hysterectomy in terms of sociodemographic characteristics and health locus of control.

## 2 Methods

### 2.1 Participants and procedure

This study adopted a cross-sectional design. A questionnaire survey was conducted among inpatients who had undergone total hysterectomy at a tertiary maternity and child hospital in mainland China. The inclusion criteria for participants were: (1) aged 18 years or older; (2) awareness of personal medical condition; (3) clear consciousness and normal communication abilities; (4) willingness to voluntarily participate in the study and sign the informed consent. Exclusion criteria were: (1) presence of other malignant tumors, severe cardiovascular or cerebrovascular diseases, or significant organ dysfunction; (2) inability to complete the questionnaire due to poor postoperative physical condition; (3) history of mental or psychological disorders. All participants were independently recruited, and each questionnaire corresponded to a single patient. Therefore, the assumption of independence of observations in subsequent logistic regression analysis was considered to be met.

Based on Kendall’s empirical rule for sample size (Ni and Chen, 2010), this study included ten independent variables, requiring a sample size of ten to twenty times the number of variables. After accounting for a 20% potential invalid response rate, the required sample size ranged from 120 to 240 cases. Previous research has suggested that the Bayesian Information Criterion (BIC) is a commonly used reference index for model fit when identifying latent classes. A minimum sample size of 200 is typically recommended for such analyses (Liu et al., 2014). Finally, 214 sample sizes were included in this study.

### 2.2 Data collection

The study was conducted from April 2022 to May 2023 in Guangzhou Provinces, China. Trained researchers visited patients on the third day after they had undergone total hysterectomy. Eligible patients were invited to participate in the study. Researchers explained the purpose and nature of the study and provided participants with the option to participate or decline. After obtaining written informed consent to participate, a demographic information sheet is distributed for collecting the demographic characteristics and clinical data and utilized tools to measure their levels of psychological flexibility and health locus of control. Each participant took approximately 15 min to complete the questionnaire. Two researchers independently reviewed all completed questionnaires to ensure data accuracy.

### 2.3 Instrument

#### 2.3.1 Sociodemographic and clinical information

Sociodemographic information was collected using the following indices: age, marital status, monthly household income, children status and menopausal status. Participants’ clinical information included malignancy status and postoperative complications status.

#### 2.3.2 Comprehensive assessment of acceptance and commitment therapy processes (CompACT)

The CompACT scale, developed and revised by Francis et al. (2016) from the UK, is used to measure the psychological flexibility of the participants. The Chinese version was translated and tested by Ge (2020), demonstrating high reliability and validity. The scale consists of 22 items, categorized into three dimensions: openness to experience, behavioral awareness, and valued action. It employs a 7-point Likert scale, with scores ranging from 0 to 6 (from “strongly disagree” to “strongly agree”), and reverse-scored items ranging from 6 to 0. Items 2, 3, 4, 6, 8, 9, 11, 12, 15, 16, 18, and 19 are reverse-scored. The total score ranges from 0 to 132. Higher scores reflect greater psychological flexibility. The Cronbach’s *α* of this scale in this study was 0.773.

#### 2.3.3 Multimentional health locus of control (MHLC)

The MHLC scale, developed by Wallston et al. (1978), was used to measure participants’ health locus of control. The Chinese version of the scale has shown a sufficiently high degree of internal consistency and reliability (Liu and Wan, 2018). It includes three subscales: internal health locus of control, powerful others health locus of control, and chance health locus of control. It consists of 20 items and utilises a 4-point Likert scale (1–4). Each subscale’s total score ranging from 6 to 36. Higher scores indicate a stronger tendency toward each locus of control. In this study, the Cronbach’s α for IHLC, PHLC, and CHLC were 0.732, 0.703, and 0.769.

### 2.4 Data analyses

Since there were no missing values in the dataset, no imputation or deletion techniques were necessary. LCA was conducted using Mplus v8.3 software. For the purposes of LCA, the continuous CompACT scores were dichotomized into a binary variable indicating low (0) or high (1) psychological flexibility. Based on previous literature (Zhao et al., 2024), we selected 4 as the median threshold, which supports the use of such a cutoff to facilitate the identification of distinct response patterns and improve the interpretability of latent class models. Specifically, scores ranging from 0 to 3 were coded as 0, representing low psychological flexibility, and scores from 4 to 6 were coded as 1, representing high psychological flexibility. Taking the new variable as the explicit indicator, the LCA of psychological flexibility was conducted by starting with the number of model classes being 1 and increasing the number of classes in turn. LCA was used to fit the model of 1–4 latent classes by robust maximum likelihood (MLR) method, and the model with the best fitting effect was determined by the model fit indices. Model fit was assessed using multiple indices: (1) Akaike Information Criterion (AIC), Bayesian Information Criterion (BIC), and sample-size adjusted BIC (aBIC). The smaller the above indexes values, the higher the model fitting degree. Besides, aBIC had a higher propensity to identify the correct classification compared to the other indices (Mori et al., 2020). (2) Entropy, which was strongly correlated with the correct classification, with higher values indicating the higher classification accuracy. When the Entropy is 0.8, it indicated that the classification accuracy exceeds 90%. (3) Lo–Mendell–Rubin (LMR) test, and Bootstrap Likelihood Ratio Test (BLRT). When the two indices reached the significant level (*p* < 0.05) at the same time, it indicated the fitting degree of the model with k classes was significantly better than that of the model with (k-1) classes (Sinha et al., 2021). Comprehensively considering the model fit indices above, we have determined the most optimal latent class model by combining the practical significance.

SPSS 26.0 was used for descriptive analysis [means and standard deviations (SD) were used to describe the measurement data, and frequencies and percentages were used for the count data]. After determining the optimal model, chi-square tests and one-way Analysis of Variance (ANOVA) were used to identify statistically significant indicators. Prior to conducting logistic regression, multicollinearity among independent variables was assessed using Variance Inflation Factor (VIF). A VIF value of less than 5 was considered acceptable, indicating no severe multicollinearity. To ensure the validity of the logistic regression model, we assessed the model fit using the Hosmer–Lemeshow goodness-of-fit test. A non-significant *p*-value (*p* > 0.05) indicates a good fit between the model and the data. Additionally, we reported the Nagelkerke *R*^2^ value, which provides an indication of the proportion of variance explained by the model. Logistic regression analysis was used to evaluate the influence of latent profiles. *p* < 0.05 indicates that the difference is statistically significant.

## 3 Results

### 3.1 Participant characteristics

A total of 216 questionnaires were distributed in this study and 214 valid questionnaires were returned, with a valid return rate of 99.1%. The participants’ ages ranged from 28 to 79 years, with a mean age of 51.12 years (SD = 8.41 years). Among them, 147 participants (68.7%) were 45 ~ 59 years old, 195 participants (91.1%) were married, 207 participants (96.7%) had children, 134 participants (62.6%) were premenopausal prior to surgery, 56 participants (26.2%) had malignant diseases, and 107 participants (50.0%) experienced postoperative complications.

### 3.2 Latent classes of psychological flexibility

**Figure 1:**
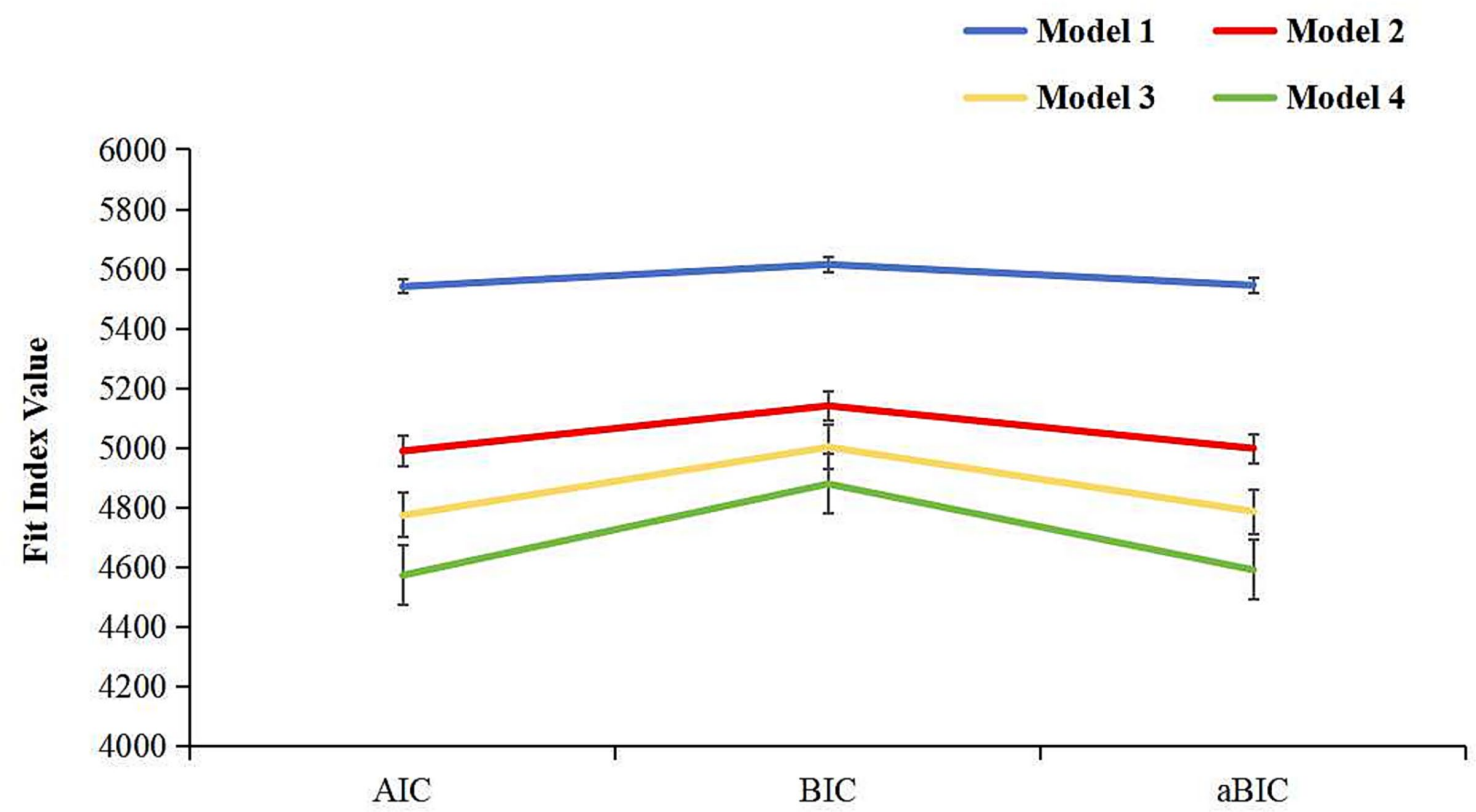
Scree plot showing the AIC, BIC, and aBIC values across latent class models.

Latent class models with one to four classes were explored using Mplus. The three-class model was selected as the best-fitting solution. It showed the smaller AIC, BIC, and aBIC values in Figure 1, significant LMR and BLRT *p*-values, and an Entropy value greater than 0.8, indicating good classification accuracy. When the two-class and three-class models were compared, both LMR and BLRT *p* values were significant. Since the k class model outperforms the k-1 class model, indicating that the two-class model was not better than the three-class model. In addition, the average posterior probabilities for the three classes were high (99.8, 99.9, and 98.3%, respectively), suggesting stable classification with low risk of overfitting. Model fit indices and classification probabilities are presented in Tables 1, 2.

**Table 1:** Model fit indices for latent classes about psychological flexibility (*N* = 214).

Model	AIC	BIC	aBIC	Entropy	LMR (*P*)	BLRT (*P*)	Category probability
1	5540.894	5614.946	5545.233	–	–	–	–
2	4989.544	5141.013	4998.419	0.941	0.015	<0.001	0.238/0.762
3	4773.670	5002.557	4787.082	0.988	<0.001	<0.001	0.673/0.107/0.220
4	4572.941	4879.245	4590.888	0.974	0.380	<0.001	0.126/0.397/0.360/0.117

**Table 2:** Average latent class probabilities for most likely latent class membership (row) by latent class (column).

Class	Inflexible Class	Moderate Class	Flexible Class
Inflexible Class	0.998	0.000	0.002
Moderate Class	0.001	0.999	0.000
Flexible Class	0.017	0.000	0.983

**Figure 2:**
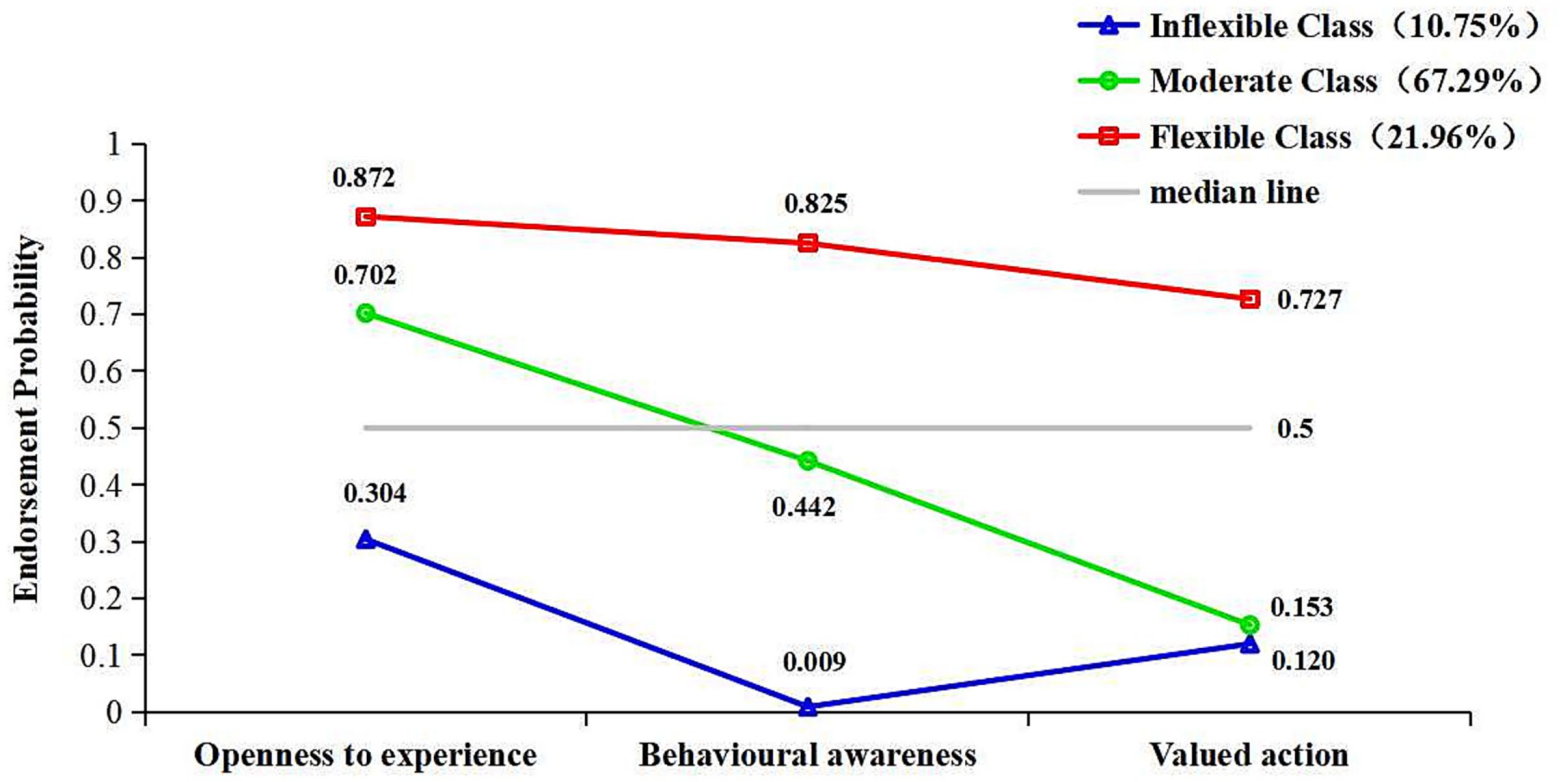
Conditional probability of psychological flexibility latent classes.

Each class was named based on the average conditional probabilities across various dimensions of psychological flexibility. The three distinct pattern classes of psychological flexibility were designated as Inflexible Class, Moderate Class, and Flexible Class. The exact responses of the three latent classes for all three dimensions of the CompACT are displayed in Figure 2.

Inflexible Class (*n* = 23, 10.75%) exhibits a generally low level of psychological flexibility, with all conditional probabilities falling well below 0.5: openness to experience (0.304), behavioral awareness (0.009), and valued action (0.120). The patients belonging to this class usually exhibit psychological inflexible, characterized by experiential avoidance, diminished behavioral awareness, and a lack of value-driven actions.

Moderate Class (*n* = 144, 67.29%) shows a moderate level of psychological flexibility, with conditional probabilities that hover around the 0.5 threshold. Their openness to experience (0.702) is well above 0.5, suggesting that they are generally open to new experiences and willing to explore different situations. However, their behavioral awareness (0.442) is just below 0.5, indicating that while they have some level of self-awareness, they may still struggle to consistently monitor and regulate their behaviors and emotions. Additionally, their valued action (0.153) is well below 0.5, suggesting that they have difficulty consistently taking actions aligned with their values, particularly in challenging situations. Overall, the patients belonging to this class demonstrates moderate flexibility, with strengths in openness to experience, but room for improvement in areas such as self-awareness and aligning behavior with core values.

Flexible Class (*n* = 47, 21.96%) demonstrates high psychological flexibility, with all conditional probabilities well above 0.5: openness to experience (0.872), behavioral awareness (0.825), and valued action (0.727). The patients belonging to this class are characterized by a high level of self-consistency, they are highly open to new experiences, maintain strong self-awareness, and consistently act in alignment with their values. These individuals are able to embrace new challenges, stay present in the moment, and make choices that reflect their core beliefs and values, even when faced with adversity.

### 3.3 Sociodemographic and clinical characteristics of the different classes

The results of the univariate analysis showed that the three classes in children status (*χ*^2^ = 9.327, *p* = 0.008) the differences were statistically significant (*p* < 0.05). There were no statistically significant differences in other demographic characteristics (*p* > 0.05). Sociodemographic and clinical characteristics of the three psychological flexibility classes are presented in Table 3.

**Table 3:** Sociodemographic and clinical characteristics of the three psychological flexibility classes (*N*, %).

Variable	Overall	Inflexible Class	Moderate Class	Flexible Class	*χ^2^*	*p*
Age					8.894	0.057
<45	35 (16.4%)	7 (30.4%)	19 (13.2%)	9 (19.1%)		
45 ~ 59	147 (68.7%)	15 (65.2%)	105 (72.9%)	27 (57.5%)		
>59	32 (15.0%)	1 (4.4%)	20 (13.9%)	11 (23.4%)		
Marital status					1.351	0.524
Married	195 (91.1%)	21 (91.3%)	133 (92.4%)	41 (87.2%)		
Unmarried/Divorced/Widowed	19 (8.9%)	2 (8.7%)	11 (7.6%)	6 (12.8%)		
Monthly household income (CNY)					1.765	0.414
<5,000	83 (38.8%)	11 (47.8%)	57 (39.6%)	15 (31.9%)		
≥5,000	131 (61.2%)	12 (52.2%)	87 (60.4%)	32 (68.1%)		
Children status					9.327	0.008
Childlessness	7 (3.3%)	2 (8.7%)	1 (0.7%)	4 (8.5%)		
Parenthood	207 (96.7%)	21 (91.3%)	143 (99.3%)	43 (91.5%)		
Menopausal status					2.950	0.229
Premenopausal	134 (62.6%)	18 (78.3%)	86 (59.7%)	30 (63.8%)		
Postmenopausal	80 (37.4%)	5 (21.7%)	58 (40.3%)	17 (36.2%)		
Malignancy status					1.120	0.571
Absence	158 (73.8%)	19 (82.6%)	104 (72.2%)	35 (74.5%)		
Presence	56 (26.2%)	4 (17.4%)	40 (27.8%)	12 (25.5%)		
Postoperative complications status					1.306	0.520
Absence	107 (50.0%)	14 (60.9%)	71 (49.3%)	22 (46.8%)		
Presence	107 (50.0%)	9 (39.1%)	73 (50.7%)	25 (53.2%)		

### 3.4 Influencing factors of psychological flexibility in different classes of participants

Before interpreting the logistic regression results, we validated key model assumptions. The Hosmer–Lemeshow test showed a non-significant result (*χ*^2^ = 76.489, *p* = 0.527), indicating a good model fit. The Nagelkerke *R*^2^ value was 0.159, suggesting moderate explanatory power. All VIF values were below 2.0, indicating that multicollinearity was not a concern in the model. Logistic regression models were developed using the latent class of psychological flexibility as the dependent variable and Inflexible Class as the control group. The results showed that compared to Inflexible Class, Parenthood (OR = 18.920, *p* = 0.033) were more likely to be classified in Moderate Class; Age<45 (OR = 0.028, *p* = 0.016) were more likely to be classified in Inflexible Class; Monthly household income≥5,000 CNY (OR = 3.795, *p* = 0.027) were more likely to be classified in Flexible Class. Multinomial logistic regression analysis are presented in Table 4.

**Table 4:** Multinomial logistic regression analysis of influencing factors affecting class of psychological flexibility (*N* = 214).

Variable	Inflexible Class VS Moderate CLASS				Inflexible Class VS Flexible Class			
	Reference: Inflexible Class				Reference: Inflexible Class			
	OR	*p*	OR 95%CI		OR	*p*	OR 95%CI	
			Lower	Upper			Lower	Upper
Age (ref:>59)								
<45	0.136	0.147	0.009	2.014	0.028	0.016	0.001	0.519
45 ~ 59	0.522	0.582	0.051	5.293	0.098	0.066	0.008	1.166
Marital status (ref: Married)								
Unmarried/Divorced/Widowed	1.763	0.558	0.264	11.777	2.156	0.454	0.288	16.130
Monthly household income (ref:<5,000 CNY)								
≥5,000 CNY	2.522	0.077	0.904	7.031	3.795	0.027	1.167	12.345
Children status (ref: Childlessness)								
Parenthood	18.920	0.033	1.277	280.414	1.088	0.939	0.126	9.377
Menopausal status (ref: Premenopausal)								
Postmenopausal	1.469	0.551	0.415	5.204	0.619	0.530	0.139	2.762
Malignancy status (ref: Absence malignancy)								
Presence malignancy	2.792	0.125	0.752	10.364	1.632	0.506	0.385	6.914
Postoperative complications status (ref: Absence postoperative complications)								
Presence postoperative complications	1.465	0.432	0.565	3.800	1.859	0.254	0.641	5.393

The ANOVA results showed that there were significant differences (*p*<0.05) among the three latent classes of psychological flexibility in relation to the IHLC (*F* = 5.649) and the CHLC (*F* = 30.810). In terms of between-group differences in IHLC, the mean score of health locus of control for participants in Moderate Class was significantly higher than that in Inflexible Class; In terms of between-group differences in CHLC, the mean score of health locus of control for participants in Flexible Class was significantly higher than that in Inflexible Class and Moderate Class. Differences in health locus of control across the latent classes are presented in Table 5.

**Table 5:** Differences in health locus of control across the latent classes (M ± SD).

Variable	Inflexible Class	Moderate Class	Flexible Class	*F*	*p*	LSD
IHLC (M ± SD)	21.57 ± 2.94	24.83 ± 4.89	23.72 ± 3.66	5.649	0.004	Moderate Class>Inflexible Class
PHLC (M ± SD)	27.13 ± 3.05	27.08 ± 3.32	26.34 ± 3.72	0.886	0.414	-
CHLC (M ± SD)	17.52 ± 3.42	17.33 ± 4.50	22.94 ± 4.04	30.810	<0.001	Flexible Class>Inflexible Class Flexible Class>Moderate Class

## 4 Discussion

### 4.1 Latent classes of psychological flexibility in patients after total hysterectomy

This study identified three distinct latent classes of psychological flexibility among patients after total hysterectomy: the Inflexible Class, Moderate Class, and Flexible Class, indicating significant heterogeneity in psychological flexibility within this patient population.

The Inflexible Class (10.75%) included the fewest participants. In the framework of Acceptance and Commitment Therapy, psychological inflexibility refers to impaired adaptive regulation of cognitive and emotional responses to changing circumstances, typically manifested as persistent experiential avoidance and disengagement from value-driven behavior (Ong et al., 2024). Patients in this class demonstrated rigid coping patterns, including emotional suppression and difficulty initiating purposeful, adaptive actions. For women undergoing hysterectomy, the removal of the uterus represents not only the loss of reproductive capacity but also potential physiological changes such as hormonal fluctuations and alterations in secondary sexual characteristics. These various stressors likely contribute to higher psychological inflexibility in this group. Clinically, this group warrants prioritized psychological support. Evidence suggests that mindfulness-based interventions can increase present-moment awareness. They also foster greater acceptance of emotional and somatic experiences. These changes may reduce avoidance tendencies and improve psychological flexibility (Masuda et al., 2022). Introducing such interventions may help disrupt rigid psychological patterns and enhance adaptive functioning in patients within this class.

The Moderate Class (67.29%) represented the majority of participants. These individuals generally demonstrated openness toward their surgical experience and emotional acceptance of bodily and psychological changes. Without well-defined personal health goals or internal motivation, they may find it difficult to translate acceptance into effective behavioral change. Motivational interviewing (MI), an evidence-based intervention approach, has been shown in prior studies to be effective in fostering behavioral change through relationship building, collaborative goal setting, motivational enhancement, and brief action planning (Cole et al., 2023). While MI has not yet been tested within this specific group, it could be a promising strategy for helping individuals in the Moderate Class convert emotional openness into concrete recovery behaviors, thereby supporting long-term postsurgical adaptation and subjective well-being.

The Flexible Class (21.96%) included patients who were able to accept themselves internally, approach their condition with rationality, and proactively engage in psychological self-regulation and action planning to manage the physical and emotional challenges of hysterectomy. Peer support programs have been shown to enhance adaptive coping in this group by facilitating mutual support, emotional expression, and experiential knowledge exchange (Grégoire et al., 2024). Facilitating interaction between patients in the Flexible Class and those in the Inflexible or Moderate Classes may strengthen social support networks while offering positive role models and practical coping strategies. Such peer-based interaction has the potential to promote a gradual shift toward greater flexibility among less adaptive patients, creating a mutually beneficial dynamic that supports recovery across all groups.

### 4.2 Differences in latent classes of psychological flexibility by sociodemographic and clinical information

Patients who had children were more likely to be categorized into the Moderate Class. In traditional Chinese family culture, children are often seen as emotional anchors and sources of spiritual support for their parents (Pan and Chen, 2022). Patients with children often benefit from a stable, supportive family environment, as this ongoing support serves as a valuable psychological resource during illness and recovery (Li et al., 2024). However, they may prioritize their children’s needs over personal health. This can interfere with rehabilitation planning and goal-directed recovery behavior. For this group, involving families in interventions can free up patients’ time for recovery and support health goal-setting.

Patients under 45 years were more likely to be classified into the Inflexible Class. The psychological impact of losing reproductive capacity is often more pronounced among younger women, who may experience heightened anxiety, low self-esteem, and emotional distress (Al-Amer et al., 2025). The uterus is not only associated with biological fertility but also symbolically linked to femininity, motherhood, and the continuity of family lineage (Li et al., 2023). Therefore, hysterectomy may be perceived as a loss of womanhood or failure to fulfill traditional family roles, especially for women who have not yet completed childbearing. These cultural expectations can intensify psychological distress and contribute to internalized stigma. Additionally, younger women may lack mature coping strategies, leading to increased experiential avoidance and reduced psychological flexibility (Lai et al., 2022). Targeted counseling and education about illness perception, with sensitivity to sociocultural values, are crucial. These measures can improve emotional regulation and adaptive functioning in this group.

Patients from households with a per capita monthly income of ≥5,000 CNY were more likely to be placed in the Flexible Class. Household income is a direct indicator of economic status, which has been shown to significantly influence subjective well-being and mental health (Sun, 2023). From a sociological perspective, patients from higher-income households experience less financial pressure and have greater access to resources and social support, which can enhance their ability to cope positively with postoperative changes (Moosa et al., 2023). In contrast, lower-income patients may face greater psychological stress. They also have limited access to healthcare resources, which can hinder recovery (Sánchez-Moreno and Gallardo-Peralta, 2022). For lower-income patients, assessing family financial situations and providing supportive psychological care can reduce emotional burden and enhance adaptive flexibility.

### 4.3 Differences in latent classes of psychological flexibility by health locus of control

This study found that post-hysterectomy patients with a stronger tendency toward IHLC were more likely to belong to the Moderate Class. These patients typically seek to manage their illness through self-control and proactive knowledge acquisition (Wallston et al., 1978). However, hysterectomy is a major traumatic event. It is largely out of personal control and can trigger severe psychological stress. In the short term, this can trigger stress responses such as emotional avoidance and maladaptive coping behaviors (Katon et al., 2020). “Active health” is an emerging concept and model for promoting holistic well-being, and the empowerment-based health education model is designed to enhance patients’ sense of responsibility for their own health, thereby facilitating behavioral change (Stepanian et al., 2023). In recent years, this model has been widely applied in postoperative rehabilitation, improving recovery outcomes by fostering self-efficacy and encouraging healthy lifestyle behaviors (Stepanian et al., 2023). Therefore, for post-hysterectomy patients with a strong IHLC, combining ACT with empowerment-based education may be particularly effective. ACT, which helps individuals build psychological flexibility, would support these patients in navigating the emotional challenges of recovery. Empowerment-based education, in turn, could reinforce their sense of control and encourage active participation in their recovery process, ultimately improving both psychological and physical outcomes.

Interestingly, patients who tended toward CHLC were more likely to fall into the Flexible Class. One possible explanation is that, for some individuals, the profound psychological disruption caused by hysterectomy leads them to seek comfort and strength from faith or other transcendent sources. Belief in spiritual or supernatural forces may provide emotional support and a sense of meaning during times of crisis (Turan et al., 2023). Patients with weaker CHLC beliefs may lack such external coping resources. This can make physical and emotional recovery more difficult. Once their internal psychological resources are depleted, they are more prone to developing patterns of psychological inflexibility (Bell et al., 2024). As the healthcare field increasingly adopts a holistic “body–mind–social–spiritual” approach, spiritual care has become an important area of research and clinical practice. Spirituality is viewed as the inner core of an individual’s life experience, and spiritual care aims to meet patients’ spiritual needs and promote inner peace and comfort (Banerjee, 2023). Studies have shown that faith, as an expression of spirituality, can help activate patients’ internal motivation and resilience in the face of illness (Zeng et al., 2024). Accordingly, integrating ACT with spiritual care practices may be particularly helpful for post-hysterectomy patients with a weak sense of CHLC. This combined approach may foster self-acceptance. It also encourages reflection on personal spiritual beliefs and enhances psychological flexibility.

Notably, this study did not find a significant association between PHLC and the latent classes of psychological flexibility. This result merits further discussion. One possible explanation lies in the cultural context of China. In general, patients tend to place strong trust in medical professionals and often regard physicians as the main decision-makers and health authorities. As a result, beliefs related to PHLC may be relatively consistent across patients, regardless of their level of psychological flexibility. This cultural tendency could reduce variability in PHLC and may help explain the lack of significant group differences. Additionally, the structured hospital environment where the study was conducted may have further reinforced a normative dependence on medical staff, limiting the expression of individual differences in this domain. Future studies could explore this further by including measures of patient autonomy or doctor–patient communication patterns.

### 4.4 Theoretical and clinical implications

From a theoretical perspective, this study contributes to a more nuanced understanding of psychological flexibility as a heterogeneous construct rather than a uniform trait. The identification of distinct latent classes supports the person-centered approach in psychological assessment and underscores the value of LCA over traditional variable-centered methods. From a clinical perspective, the Inflexible and Moderate Classes accounted for 78% of the sample, indicating substantial room to improve current ACT-based interventions for post-hysterectomy patients. In particular, patients in the Inflexible Class require focused clinical attention. Support from ongoing care, family involvement, and available resources can improve psychological flexibility and further promote overall recovery. Additionally, promoting interaction with patients in the Flexible Class can strengthen social support and provide adaptive role models, supporting the progression of Inflexible and Moderate Class patients toward greater psychological flexibility, resulting in mutually beneficial outcomes.

### 4.5 Limitations

This study has limitations. First, this study was a cross-sectional design. Future research should consider conducting longitudinal analyses across different stages of the hysterectomy process to explore the dynamic evolution of psychological flexibility over time. Second, as the data were collected through self-report questionnaires, there is a potential risk of response bias. Given the sensitive nature of reproductive health and the cultural perceptions surrounding hysterectomy, participants may have been influenced by social desirability, leading to underreporting of psychological distress or overreporting of positive adjustment. Additionally, more in-depth investigation into other relevant influencing factors is warranted to provide stronger empirical support for the development of targeted and precise clinical interventions. Furthermore, future research should consider conducting intervention trials tailored to the psychological profiles identified through latent class analysis. These interventions may include ACT-based programs or empowerment-oriented approaches. Such trials would provide empirical evidence on the clinical relevance of psychological flexibility classifications in shaping post-hysterectomy psychological outcomes.

## 5 Conclusion

This study found that psychological flexibility among post-hysterectomy patients remains suboptimal. Through LCA, three distinct psychological flexibility profiles were identified: the Inflexible Class, the Moderate Class, and the Flexible Class. These findings highlight the need for early psychological assessment prior to surgery, allowing healthcare providers to identify patients in the Inflexible Class and Moderate Class based on their specific characteristics and offer tailored care accordingly. For patients in the Flexible Class, peer support initiatives can be encouraged, enabling them to share positive coping strategies with others and foster mutually beneficial outcomes. For those in the Moderate Class, integrating ACT with empowerment-based educational models may improve treatment adherence and promote proactive engagement in recovery. Most critically, patients in the Inflexible Class require focused attention. For this group, a combined approach involving ACT and spiritual care is recommended to help them better adjust to the emotional and physical challenges of hysterectomy. In conclusion, healthcare professionals should develop and implement targeted intervention strategies based on the distinct psychological flexibility profiles identified in this study. Doing so may enhance the effectiveness of care and promote overall well-being among patients undergoing total hysterectomy.

## Data Availability

The original contributions presented in the study are included in the article/supplementary material, further inquiries can be directed to the corresponding author.
